# Building Responsive Intersectoral Initiatives for Newcomers in Toronto: Learning from Service Providers’ Experiences in the Context of COVID‑19

**DOI:** 10.5334/aogh.4583

**Published:** 2025-01-22

**Authors:** Carly Jackson, Shinjini Mondal, Erica Di Ruggiero, Lara Gautier

**Affiliations:** 1 Dalla Lana School of Public Health, The University of Toronto, Canada; 2 École de santé publique, Université de Montréal, Canada; 3 Centre de recherche en santé publique, Université de Montréal and CIUSSS du Centre‑Sud‑de‑l'Ile‑de‑Montréal, Canada; 4 SHERPA University Institute, CIUSSS du Centre‑Ouest‑de‑l'Ile‑de‑Montréal, Canada

**Keywords:** Refugees, asylum seekers, migrants without status, intersectoral collaboration, community organizations, COVID‑19

## Abstract

*Background:* Newcomer populations in urban centers experienced an exacerbated effect of coronavirus disease 2019 (COVID‑19) due to their precarious living and working conditions. Addressing their needs requires holistic care provisioning, including psychosocial support, assistance to address food security, and educational and employment assistance. Intersectoral collaboration between the public and the community sector can reduce vulnerabilities experienced by these groups.

*Objective(s):* This research explores how community and public sectors collaborated on intersectoral initiatives during the COVID‑19 pandemic to support refugees, asylum seekers, and migrants without status in Toronto, Ontario, Canada to generate lessons for a sustainable response.

*Methods:* The research uses a participatory governance approach to study multiple qualitative cases (with a case being an intersectoral initiative). We conducted interviews (*n* = 25) with community and public sector frontline workers and managers, as well as municipal/regional/provincial policymakers and funders. The data were analyzed thematically with an inductive approach.

*Findings:* The analysis covers four key themes: (1) vulnerable newcomers’ circumstances regarding accessing the social determinants of health during COVID‑19; (2) the process of designing specific interventions to target these populations’ needs and service access challenges in the context of COVID‑19; (3) the implementation phase of the initiatives, including any associated challenges and lessons learned; and finally, (4) long‑term potential sustainability of the initiatives.

*Conclusions:* The findings demonstrate that intersectoral initiatives can be implemented to develop a responsive service for marginalized populations; however, their translation beyond pandemic settings would require institutional mechanisms to bring policy shifts to provide a bottom‑up collaborative approach.

## Introduction

The coronavirus disease 2019 (COVID‑19) pandemic has highlighted the preexisting inequities in modern societies [1]. A significant body of research conducted by scholars and by civil society has highlighted that ethnoracial populations were at a considerably higher risk of contracting the disease and having adverse effects; additionally, access to social determinants was strongly linked with COVID‑19 outcomes in these populations [2]. The migrant population formed a particularly made‑vulnerable group, both from the pandemic’s direct and indirect effects due to their often limited access to health care and their precarious economic and social status, which was affected by their living and working conditions, limited knowledge of their rights and access to networks, and cultural and linguistic diversity [3, 4]. The high cost of living and lack of accessible and affordable housing in Canada’s major cities further compounded these conditions [5]. However, migrants are not a homogeneous group, and thus, they have been differentially impacted during the pandemic [6].

The social determinants of health for migrants are complex and interrelated, and their experiences are heterogeneous, requiring intersectoral responses. Migration itself makes the person more vulnerable; additionally, they may be socially and economically more disadvantaged compared with the host community, leading to a lack of social cohesion and difficulties in accessing information on health and social services [7]. These challenges are likely to vary across urban and rural settings. While migrants settling in major cities in Canada may face less social exclusion due to a higher density of diasporic communities, the higher cost of living and lower availability of affordable housing characteristic of larger cities have been reported to further heighten their vulnerability [8].

In her report on public health post‑COVID‑19, Canada’s Chief Public Health Officer highlighted the need for collaboration and a coordinated response across agencies and organizations at all levels [9]. In Canada, various provincial and federal programs aim to support migrants in accessing health care and social services. However, multiple barriers to access remain [10]. In the context of COVID‑19, surveillance data indicate that in larger cities, refugees, asylum seekers, and migrants without status have been disproportionately affected due to their interrelated socioeconomic factors [11, 12]. For example, Xia and colleagues (2022) demonstrated that higher incidences of COVID‑19 occurred in cities with higher proportions of visual minorities, recent immigrant populations, high‑density housing, and essential workers [12]. Furthermore, research in urban settings in Canada suggests that COVID‑19 initiatives related to access to testing have been challenging for migrants because of their vulnerabilities related to class, culture, and economic status [13].

Given the health disparities experienced by newcomer populations during COVID‑19, community engagement and mobilization have been key for intersectoral collaboration and one of the pillars in the pandemic response through providing responsive and transparent services that cater to the diverse needs of vulnerable and marginalized groups [14]. A recent rapid review by Loewenson and colleagues (2021) identified the critical role played by community organizations in implementing rights‑based, equity‑driven public health interventions [15]. However, more evidence is required to understand, document, and report the dynamic nature of intersectoral collaboration, especially in COVID‑19.

Intersectoral action recognizes the influence of social, cultural, political, and economic factors on health and that engagement with other sectors is imperative to developing a holistic response that is sensitive to the needs of the target population [16]. An intersectoral approach can promote health equity through seeking collaboration and partnership among different partners [17]. This approach suggests that improving health, well‑being, and health equity requires collaborative efforts between different sectors of society, e.g., from different levels of government, types of organizations, and community bodies [17–19]. Previous research has provided evidence of coordination between health and social services for refugees, which contributed to improved quality and effectiveness of care received [20]. Moreover, some evidence suggests intersectoral partnerships influence the creation of health policies that improve social and physical environments for racialized and marginalized groups of people [21, 22].

Despite the potential benefits and increased calls for improving intersectoral action, services remain fragmented and difficult to access, especially for marginalized populations. In response to this gap, our research focused on understanding the experiences of service providers, funders, and decision‑makers in designing, implementing, and sustaining responsive services through intersectoral collaboration for newcomers during the COVID‑19 pandemic in Toronto, Ontario. As one of Canada’s most populous provinces, Ontario receives the largest share of refugees, asylum seekers, and migrants without status [23]. Indeed, between 2016 and 2021 the number of external migrants—meaning those arriving from outside of Canada—arriving in Toronto was 242,185 [24]. Moreover, one in seven external migrants to Canada settled in Toronto, making Toronto an important site of inquiry [24]. Due to reporting challenges, it is difficult to ascertain the proportion of these external migrants that would fall into the migrant classes of interest in this research, as the difference between asylum claims versus approvals is not always reported specific to individual cities and the number of migrants living without status is notoriously difficult to track. However, between 2016 and 2021 approximately 17.7% of migrants in Toronto arrived with approved refugee status. This amounts to approximately 42,866 people over this 5 year period [24]. This number is likely higher when considering asylum claimants and migrants living without status in the city. In this research, the term “vulnerable newcomers” was defined as asylum seekers, refugees, and migrants without status who have been in Canada for less than 5 years. Using a participatory approach, we focus on intersectoral collaborations between public and community actors, highlighting critical considerations for future policies and programs for vulnerable newcomers in Toronto, Ontario and Canada more broadly [25].

## Methods

This analysis is part of a larger multiple‑case study examining the responsiveness of COVID‑19 services for vulnerable newcomer populations in three cities in Canada: two large Canadian cities, Toronto and Montreal, and a smaller city with a history of high volumes of diverse newcomer populations, Sherbrooke. This present study reports on data from the Toronto portion of the study. The larger research project used a participatory governance approach in which the research team collaborated with knowledge users (i.e., refugees, asylum seekers, community‑based organizations [CBOs], public organizations, and policymaking bodies) throughout all stages of the research and as cocreators of any research products [25]. An early step in the research actively engaged knowledge users in deliberative workshops to compile and examine key initiatives developed by both community‑based organizations and public organizations and aimed at addressing the consequences of the pandemic. A total of eight intersectoral initiatives were selected and then grouped into two categories: (1) interventions targeted at dealing with the health consequences of the pandemic (i.e., vaccination programs) and (2) initiatives targeted at the social determinants of health impacted by the pandemic (e.g., food insecurity, income support, virtual care, etc.) (see Table 1 for an overview of the selected initiatives). To protect the anonymity of participants, we have utilized broad descriptors of the initiatives and the partner organizations. For more detailed information on the methods of the broader project, see Gautier et al. (2023) [26].

**Table 1:** Key Selected Initiatives.

INITIATIVE CATEGORY	NAME OF INTERVENTION	OBJECTIVE	KEY PARTNERS
Initiatives against COVID‑19	Vaccine Clinic Initiative	Reduce anxiety and vaccine concerns; multilinguistic vaccine information support	Community and academic organizations
	Community Volunteers Initiative	Increase vaccine confidence, access, and uptake	Community, city, and municipal organizations
	Vaccine Promotion Initiative	Improve vaccine‑related communications	Community and local health organizations
	Vaccine Access Initiative	Securing vaccine appointments, multilanguage support, and volunteer support	Community and tertiary care organization
Initiatives targeted at the social determinants of health impacted by the pandemic	Access to Food Initiative	Provide nutritious and healthy meals	Community, federal, and city level organizations
	Financial Awareness Initiative	Navigate government pandemic support programs	Community and federal organizations
	Virtual Care Initiative	Build healthcare staff capacity to prioritize client’s needs	Community and local health organizations
	Volunteer Integration Initiative	Social and integration support	Community and tertiary care organization

### Recruitment

After obtaining ethical approval from both the University of Montreal (certificate no. 2022‑1626) and the University of Toronto’s (protocol no. 00041815) Research Ethics Offices, we sought to recruit key informants with experience in designing and/or implementing the selected initiatives in Toronto. We recruited frontline workers, managers in CBOs, policymakers, and funders at provincial and municipal levels who participated in the various selected initiatives. Utilizing purposive sampling, potential participants were then invited to participate in an interview [27]. Snowball sampling was also employed through recommendations from participants of additional potential participants who would be a good fit for the project [28].

### Data collection

Semistructured interviews were conducted in English from June to September 2022, over the digital meeting platform Zoom, using a semistructured interview guide. The guide was developed after a thorough review of the literature on complex adaptive systems research in the health and social services literature, as well as material related to coevolution, intersectoral action, and health system responsiveness. The interview guide elicited the health and social services provided to newcomers, the impacts of COVID‑19 on organizational practices, the emergence and implementation of cross‑sectoral partnering and dialogue, institutionalizing the initiatives, and, finally, issues regarding the sustainability of the initiatives (see Appendix A for a list of example interview questions and subprompts). As we were interviewing across a range of initiatives and professional roles of key informants, there was some variation in interview guides to allow them to the specific to the participant. The example questions provided in Appendix A capture the essential elements probed in each interview. Each interview ran for approximately 60–75 min. Participants were not compensated for their time in participating in an interview as they were all service providers.

### Analysis

All interviews were digitally recorded and transcribed verbatim. As this portion of the project examined only a subset of the data, a separate thematic analysis was most appropriate to understand these data in isolation from the larger data set [29]. An inductive approach to thematic analysis was utilized to create a comprehensive codebook to identify, analyze, and subsequently report key themes from the data [30]. The comprehensive codebook was created by two research team members (C.J. and S.M.) after a thorough reading of all the transcripts, which then informed the analytic framework. Using the codebook, all the transcripts were carefully coded by two members (C.J. and S.M.) of the research team. Regular check‑in meetings were held between the two coders to ensure consensus and inter‑rater reliability.

## Results

In total, 25 key informants were interviewed in Toronto (see Table 2 for an overview of key informant categories). Due to recruitment challenges, interviews with frontline workers and community‑based organization managers disproportionately represented initiatives targeted at COVID‑19 (e.g., vaccination programs). However, interviews with city managers, policymakers, and funders covered a broader range of the selected initiatives, including both the direct impacts of COVID‑19 and the larger consequences of the pandemic (e.g., food insecurity, income assistance, settlement services, etc.).

**Table 2:** Key participant categories.

PARTICIPANT CATEGORIES	
Frontline workers	3
CBO managers	14
Policymakers/funders	8
Totals	25

Our analysis covers four key themes in the trajectory of the initiatives studied: (1) vulnerable newcomers’ circumstances regarding accessing the social determinants of health and the ways in which COVID‑19 exacerbated their challenges; (2) the process of designing specific interventions to target this populations’ needs and service access challenges in the context of COVID‑19; (3) the implementation phase of the initiatives, including any associated challenges and lessons learned; and, finally, (4) long‑term potential sustainability of the initiatives. Each of these themes will be examined in more detail throughout this section.

### COVID‑19 and the exacerbated hardships for vulnerable newcomers

Participants across all categories emphasized that vulnerable newcomers in Toronto faced inequities in accessing the social determinants of health before the COVID‑19 pandemic and, critically, that the pandemic conditions further entrenched these challenges. For example, when discussing social isolation experienced by newcomer populations during the pandemic, this CBO manager described how some *“specific factors for that extreme social isolation were lower language levels, lack of access to language courses, which are the very first way that most newcomers are brought out of their homes and into community with other newcomers. And so, without those language classes, or other employment training classes, or opportunities for in‑person learning, refugee newcomer households, were just extremely isolated.” (participant no. 2, CBO manager)*. As highlighted by this participant, language learning opportunities provide key entry points for newcomers into Canadian society. In emphasizing the trickle‑down impact of these lost opportunities, this participant further highlighted how the pandemic *“kind of slowed down opportunities for integration, because, if you can’t learn to speak English, you can’t find work. You can’t connect with your community, you can’t volunteer, it’s extremely difficult to integrate.” (participant no. 2, CBO manager)*. Indeed, inequity in accessing the social determinants of health during the pandemic ranged across language learning, access to jobs, and even access to healthcare services and COVID‑19 vaccinations.

According to many participants, many individuals living in Canada during the COVID‑19 pandemic experienced challenges with job loss, housing and food affordability, and social isolation; however, these challenges were more acutely felt by the newcomer population due to preexisting barriers. As one participant described the pandemic *“put a lot of hardships on our families and having to get back out there and secure a job, secure income, whether or not they were coming from something that they were doing night shifts and now they have to do day shift, but now the children are home, so it’s become very difficult.” (participant no. 16, CBO manager)*. In addition, the stress and difficulty surrounding job loss, lack of childcare, and the generally high cost of living in a city such as Toronto on often inadequate salaries led to *“an increase in the domestic violence within our residents in general, a lot of these being newcomers and I think out of the pandemic with the loss of jobs, this obviously affected several residents in terms of income, children having to be home.” (participant no. 16, CBO manager)*. Indeed, some of the most reported challenges COVID‑19 imposed or exacerbated for the newcomer population included an increased incidence of domestic violence, lack of childcare, higher rates of job loss, food and income insecurity, and challenges accessing culturally or linguistically sensitive healthcare services, especially COVID‑19 services such as vaccination.

### Designing interventions for vulnerable newcomer populations

A growing understanding among key informants that the pandemic was exerting heightened pressures on vulnerable newcomer populations in Toronto led to a call to design initiatives to tackle the pandemic’s health and social impacts while keeping the specific needs of these communities in mind.

#### Understanding community need

One of the first challenges participants reported for newcomers was the global shift to virtual service delivery. While organizations were rapidly pivoting to online formats and virtual services, participants reported needing to consider challenges that may be experienced by the newcomer population. Such challenges included potentially lower digital literacy, lack of access to telecommunications technologies (e.g., computers, smartphones, and internet), and lack of private spaces to receive services digitally at home. In accounting for these challenges, one participant highlighted the importance of meeting newcomers at their specific comfort level with technology, *“so that could be like a video meeting over WhatsApp, it could be a Zoom meeting, it could just be a phone call… that really depended on the newcomers’ comfort level with technology.” (participant no. 2, CBO manager)*. While another participant pointed to the expense and privacy challenges virtual care delivery can impose, as *“internet is expensive. And so, for many immigrants and refugees, utilizing virtual care meant that they would have to go to a library, or they would have to go to a friend’s house. Now, there’s nothing wrong with that, in and of itself, you’re getting access to care. But it raises issues around privacy, comfort.” (participant no. 21, CBO manager)*. These participants highlighted the importance of considering the unique social contexts experienced by newcomers when designing an initiative to holistically address the community’s needs.

In other cases, participants noted that being responsive to newcomer needs could be accomplished within existing programs. One participant involved in a vaccination initiative described how the initiative was able to account for newcomers’ language needs by instructing staff at the clinic to utilize mobile interpretive services. Each day before the clinic opened, she would simply remind her staff, *“here’s the number for interpretive services, here’s [organization]’s account number, here’s what you say, steps 1–2–3–4.” (participant no. 10, CBO manager)*. The organization had set aside a portion of the budget for the clinic for interpretation services, and by being mindful of linguistic challenges that may impede the newcomer population from becoming vaccinated, the initiative succeeded in considering the needs of service users without a radical redesign or significant additional costs.

#### The need for a rapid and adaptive response through intersectoral collaboration

To adapt to the unprecedented and rapidly developing COVID‑19 situation, participants discussed the need to quickly design initiatives with limited resources and limited information about COVID‑19. However, many participants highlighted that this was largely facilitated by the unifying force of COVID‑19. As explained by this policymaker, *“COVID allowed us to have one common goal or one common enemy or whatever you want to call it.” (participant no. 5, policymaker)*. As the threat of COVID‑19 brought together various organizations, a rapid and adaptive response was facilitated in designing the various initiatives.

### Implementing interventions for vulnerable newcomers: facilitators and challenges

The implementation phase of the initiatives utilized and strengthened any prior existing relations and created new partnerships. The alliances among city‑level and more prominent or well‑funded organizations ensured enough resources, whereas collaboration with smaller community‑based organizations ensured broader outreach and facilitated more equitable access to services by the under‑served newcomer population.

#### Enhancing coordination through formal coordination and informal cooperative relationships

Participants shared examples of willingness to not only work within their own organization but also with external organizations to share knowledge and support the holistic needs of the population. As described by one policymaker, *“it’s really important to us that this information doesn’t sit and stay exclusively within the public health agency. So, beyond our external collaboration, we work with other departments. So, we have engaged with [Federal Department 1], we’ve also engaged with them on their migration and health team to share best practices and talk about key learnings and overlap between our projects.” (participant no. 18, policymaker).* Moreover, while explaining the partnership across organizations to implement joint programs, the manager from one community‑based organization explained that any commitment that they will continue to provide services during the pandemic was facilitated through working together, as *“The basic building block is we’re partners, you’ve got vulnerable clients who need access we had to make joint decisions about what would we offer, how would it be offered? When would it be offered? Where would it be offered? We had to have clarity around why we were offering it. We had to make sure that everybody had that basic commitment too; we’re going to remain open, and we’re going to remain accessible as a part of their service, as well city wide.” (participant no. 23, CBO manager).* As this participant made clear, while partnerships are essential, a clear delineation regarding roles and responsibilities was also essential to the success of the initiative.

Building partnerships both between organizations and with the newcomer community was a critical factor in the pandemic to address equity concerns. Participants emphasized that community engagement and establishing trust with the newcomer community was necessary, and hence, many city‑level and health‑based organizations partnered with community‑based organizations working with equity‑denied groups. Sharing the example of a vaccine program, a policymaker shared the importance of engaging communities as *“there was testing hesitancy, primarily amongst racialized populations and vulnerable populations and the homeless. We were doing testing with our partners in shelters, in group homes, to reach the most vulnerable members of our community. So, we had built those relationships. And when the vaccine came, then things really expanded in terms of really needing to do that community outreach to vulnerable populations to help them get vaccinated, but we have the relationships already” (participant no. 17, policymaker)*. Our participants emphasized that this trust‑building was largely developed through informal actions with the community and leveraging any preexisting relationships the community‑based partners may have already had with the newcomer community.

While stressing the importance of developing formal relationships, one of the managers from the community organizations also mentioned the role of developing personal and informal relationships. At the height of the pandemic, when the response had to be rapid, it was quicker and more efficient to use their informal relationships. The participant further explained that even in cases of a formal agreement, they often use their informal relationship to enable the work *as “those personal relationships are so important. And even when there are institutions and formalized arrangements, and expectations that are documented, it’s often through those individual relationships that that information is more readily shared and, and things get done.” (participant no. 15, CBO manager)*.

While informal relationships are key, the importance of formal city‑wide coordination mechanisms cannot be ignored. Policymakers shared the example of a task force set up during COVID‑19 that enabled coordination, data pooling and review, knowledge sharing, and rapid response.

#### Leadership by key actors

Several participants reported that city‑level and local organizations played a key role in leading the response. One of the policymakers expressed that leadership by community organizations in tailoring and building the program and services appropriate for their community was helpful due to their understanding of the needs of these often hard‑to‑reach populations. This also led to a change in approach to delivering services during COVID‑19, as traditionally, the user comes for services, but given the vulnerability and access issues, services were designed to be delivered door to door, which led to an increase in rates of testing and vaccination. As explained by this policymaker, *“I think we saw the value in going to sort of where people were, so whether that’s apartment buildings and doing door knocking and in offering vaccines or going to churches, going to different places, basketball courts the streets, TTC stations, you know, the Community Ambassadors really informed where we might think about going in terms of offering vaccine in a way that was convenient for the people that needed them.” (participant no. 5, policymaker)*. The study participants also highlighted the role of individual leadership within organizations, who spearheaded and forged formal and informal relationships across different organizations.

#### Funding mobilization and human resource challenges

During the pandemic, many CBOs received additional funding to design programs for marginalized people, which enabled the hiring of required staff and their training. As this policymaker described, *“there was also a lot of, I would say, resources that were, that was developed by [municipal health governance agency] and other sort of health service providers in the materials that were needed to promote vaccines.” (participant no. 5, policymaker)*.

Although funding support enabled the creation of new programs, even for already existing programs, there was a considerable additional cost involved in the move to virtual service delivery and thus posed significant implementation challenges. The additional costs were towards enabling the provision of computer and information technology (IT) costs, subscription of online services, creation of additional space, and staff training and development. Participants discussed the benefits of resource pooling as a way of overcoming some of these challenges through achieving a higher pool of funds to cover a larger client base and minimize waste.

Many participants also spoke about the associated challenge of recruitment, deployment, and burnout in staff during the pandemic. Pointing to the burnout in staff, one of the managers suggested that networking with the newcomer population has been reduced because of the nature of the pandemic and staff burnout. As the manager described, *“there is no time, there are only eight hours in a day, also staff burnout as well. Instead of providing services that are much needed, talking about the clients that we serve, and instead of focusing on trauma‑related issues and things like that, if we do only outreach, the bigger issue of the wellbeing of the individuals is going to be affected.” (participant no. 13, CBO manager)*. This reduced their capacity to focus on well‑being, where they needed to talk to their clients about trauma and provide them with access to information to make informed choices.

Another challenge managers faced was redeployment. Many projects initiated during the pandemic had shorter funding cycles that did not allow for hiring permanent human resources, so organizations either deployed resources from other programs or used volunteers to sustain the human resources.

### Sustaining initiatives beyond the pandemic and the associated challenges

When asked about sustaining the interventions beyond the pandemic, many participants agreed that many elements of the programs during COVID‑19 should continue. The programs focused on building vaccine confidence in the newcomer communities and creating trust in the community; these relationships can be used for addressing other forms of vaccine‑preventable diseases. These programs also built capacities among community workers and volunteers to develop trustworthy relationships in the community and can be leveraged for other health promotion programs. As described by one CBO manager, *“that’s it’s all about immunization… And then you have even the pre‑pandemic fears about whether these vaccines are impacting children’s health and development. And there’s a lot of information and misinformation going around these days that needs to be addressed. So, part and parcel of keeping this kind of project alive is to help people navigate those fears and concerns and give them accredited information that they can rely on and trust and have it come from a source that would be already trusted.” (participant no. 8, CBO manager)*. Another manager from a community organization shared the example of collaboration between provincial, municipal and community organizations to increase the outreach and scope of programs to the newcomer population. An enhanced culture of collaboration enabled fundraising, system navigation, and door‑to‑door outreach for the newcomer population, which was essential and needed to be continued beyond the pandemic.

The most reported challenge was the limited funding envelope, which was only for the pandemic and gradually tapered off with each wave, making it difficult to sustain the programs. However, a provincial policymaker highlighted that the pandemic shifted and changed the usual siloed approach of departments and organizations, as it required joint efforts to initiate a swift and responsive approach for newcomers and made the argument for continuing a partnership approach to work.

One of the managers from the local health unit described multiple pathways to sustaining initiatives beyond the pandemic as a spiderweb and not a mere financial one. She explained that the large programs that are run citywide are complex systems to manage; a mere scaling down or smaller scope may need to transition into a completely new model and may not have components of the original program. The second approach to program sustainability would be incorporating the learnings into an existing model, where some of the newly hired staff can be retained, but that would depend on the social issue the program is addressing and how much upscaling of the skills is required in the new program. For example, as the manager further explained, *“in a lot of the other social issues [it’s] an easy fix because we’re looking at very intricate problems. That requires a skill level of education and lived experience like that.” (participant no. 25, health unit manager)*.

## Discussion

COVID‑19 acted as a common crisis that spurred the need for not doing business as usual and was the catalyst for collaboration among our study participants. Similar to other recent academic work in this field, this present study confirmed the need for taking a different approach to health equity for vulnerable migrant populations [31–33]. The status quo of a siloed approach to service delivery—i.e., where the health sector deals solely with health, while the community sector deals solely with the social determinants of health—does not adequately address the needs of these communities, especially in times of health emergencies. As such, intersectoral action between these sectors is critically needed, and as demonstrated by our present study, the collaborative spirit ought to be bolstered by the need to provide a rapid and holistic response, to share materials and information to prevent duplication, to share best practices, and to work with partners and organizations with trustworthy relationships with marginalized populations. Through this research, we documented and analyzed initiatives that emerged during COVID‑19 through a wide variety of collaborations between partners. While some organizations collaborated with federal or provincial agencies, others collaborated with public organizations and community‑based organizations to respond acutely to the needs of the vulnerable newcomer population. While the partnerships varied in format, every partner felt the need for collaboration, as they often found their solo response was not comprehensive and lacking, whether that be from inadequate funding, a lack of preestablished trusting relationships with the community, staffing shortages or burnout, or any other challenge.

The newcomer population presents unique considerations when designing and implementing health and community services. For example, any equitable service ought to require sensitivity to cultural and linguistic diversity, which, in turn, builds trust and confidence in newcomers. Indeed, a systematic review focusing on delivery models shared that access can be improved in the refugee population by multidisciplinary staff, use of interpreters, transportation, and appointment booking navigation. The present study also highlights that providing culturally sensitive care could improve the quality of these services [20]. Moreover, our study similarly demonstrated that cultural sensitivity was significant in building vaccine confidence, as it was critical to tailor the delivery to meet social, cultural, and linguistic needs.

The diversity in the needs of newcomers also highlights the importance of equity issues. The service providers in the study highlight the approach of avoiding a “one‑size‑fits‑all” approach and instead developing a “hyper‑local” response to cater to the needs of diverse groups. Building a tailored response often requires engaging communities in long‑term partnerships, as building trust takes time, and, especially in the context of a health emergency where time is of the essence, the organizations that had previously invested in cultivating these relationships could more efficiently tailor the initiatives and develop a timely response. It is common practice for community‑based organizations to work with community mobilizers or volunteers from that community who have knowledge and trust in the community. For example, a multicountry study, including Canada, by Berardi and colleagues (2020) highlighted the importance of community engagement and dialogue as key to addressing vaccine equity [34]. It also highlighted the changes that must be considered in policy environments as migration has intertwined complex vulnerabilities [34]. Furthermore, another study in Toronto also found that social connections and relationships between communities and formal institutions were facilitators in providing an equitable response in the context of COVID‑19 [35]. Therefore, informing initiatives and decision‑makers of the lived experiences of affected newcomer communities can help prioritize the dismantling of vulnerabilities experienced by these communities, such as stigmatization and structural racism [36]. This present study demonstrated similar themes regarding the importance of community engagement and trust‑building as key building blocks in providing timely and effective services that specifically address the needs of these populations.

The pandemic provided opportunities for coordination by providing additional funding opportunities that increased collaboration between different organizations, especially with CBOs, which have the expertise to engage with the newcomer community and, often, already have existing relationships with the newcomer community. The swiftness needed to adapt initiatives and share information led to the creation of formal and informal ways of coordinating. However, beyond the crisis context, how to sustain and draw learnings from these initiatives remains a big question. While the initiatives, as they were designed in the pandemic context, would not be appropriate to sustain in their original format, there are still lessons that can be drawn from the experience. Most important is investment in building partnerships and relationships with the CBOs to better accommodate leveraging the coordination mechanisms that allowed sectors to move out from their silos. Second, the success of peer engagement in newcomer populations within intersectoral initiatives cannot be overshadowed. Across the initiatives we examined in this study, the use of community volunteers and workers continued to be one of the most recognized success factors in reaching newcomer populations on a level they could relate to. These community volunteers were often previous migrants who were culturally, linguistically, and/or spiritually similar to the communities that the services were attempting to reach. As such, they were more relatable and had an enhanced level of authority and trust in the community. Through this peer engagement, these services saw higher levels of uptake of services, and importantly, in the context of COVID‑19, a higher uptake in vaccination and testing. Without the use of community volunteers, it is unclear if COVID‑19 services would have had the reach that they did, and as such, the usefulness of peer engagement ought to be a key lesson learned on how to engage with vulnerable communities moving forward for both routine health and social services (e.g., for other public health campaigns) or in the context of a future health emergency.

### Limitations

No study is without limitations. First, this study highlights the emergence and implementation mechanisms of the selected initiatives, and thus, it only reports data from the perspective of service providers, funders, and policymakers. The larger study captured service users’ perspectives, but they are to be published separately and are not shared in this publication. As such, any descriptions of the hardships or experiences of vulnerable newcomers or how the selected initiatives were appreciated by newcomers are from the perspective of service providers based on their experiences working with these populations. The second is the time frame in which we were conducting the research. We conducted the project during the pandemic, and we needed to be sensitive to the high‑intensity workload of service providers during this time. Sometimes, even after repeated follow‑ups, it was challenging to schedule an appropriate time for an interview. Although we revised our overall timeline of data collection to be more sensitive and inclusive, sometimes, despite our efforts, we were not able to schedule time with the designated; we then asked for an alternate contact who would be able to spare time. Finally, we were also limited by the high turnover of staff during the pandemic period. The initiatives we studied in this project had been initiated during the pandemic, and due to staffing shortages, the initiatives often had to borrow staff from other programs while the program was getting off the ground. By the time we had contacted them for an interview, the staff had often been reassigned back to their original positions or had left the organization entirely and, in turn, were unavailable for an interview. This was truer for frontline staff than for managers. Conversely, some of the participants we interviewed had only joined the initiative in its later stages and, thus, could only comment on their experiences with the initiative after they began working on it.

## Conclusions

The COVID‑19 experience in Toronto demonstrated that well‑funded intersectoral engagements can develop a responsive service to marginalized populations, such as refugees and asylum seekers, especially in times of crisis. There is an opportunity to capitalize on the relationships and trust developed through engaging in collaborative intersectoral partnerships. It is also clear that policymakers, funders, community‑based organization managers, and frontline workers agreed on the need for constant impetus toward stronger implementation of intersectoral collaboration. The research participants agreed on a need for both a bottom‑up approach of leveraging preexisting partnerships and building on preestablished trust, as well as needing sustained sources of stable funding mechanisms and conducive policy environments at the federal and provincial levels that enable shared leadership and decision‑making. The challenge remains on how to implement additional lessons learned from the pandemic, how to shift from usual ways of working, and, most importantly, not return to siloed approaches to community health. However, it is clear is that bringing institutional mechanisms and broader shifts in policies that provide a nurturing space for a bottom‑up collaborative approach that goes beyond crises will be critical to health emergency management in the future.

## Data Availability

Through our informed consent process, the participants of this research were promised absolute confidentiality of their interview data. As such, the individual transcripts are not available in any qualitative data repository. The datasets used and/or analyzed during the current study can be made available from the corresponding author on reasonable request.

## Appendix A: Example Interview Guide

- **Background questions**
  - Please tell us about your current position. Which sector do you work in?
  - Please tell us about your organization and its scope of work. In which neighborhoods of Toronto is your organization located?
  - How many months/years have you been in this position? Can you tell us about the activities your role engages in?
    Probe: participation in the governance of collaborative networks involving the social/community/health sectors
- **Health and social services provided to newcomers**
  - Can you share about the living conditions of vulnerable newcomers (i.e., refugees, asylum seekers, undocumented workers…)?
    Probe: What are their needs? How has it changed during different stages/waves? Were there any perceptible changes since the beginning of the pandemic?
  - What are the current problems/challenges with access to health and social services?
    Probe How can we ensure the continuity of support services (community, health and social services)?
- **Cross‑sectoral programs‑design, implementation, and practices**
  - During the COVID‑19 pandemic, several promising initiatives have been implemented. I would like to tell you about an initiative in which you participated/contributed, the XXX initiative, deployed in the X,Y,Z neighbourhood(s) of Toronto. Which problem(s) did this initiative tackle? How did the various solution scenarios emerge?
  - What influenced the decision to implement this initiative? What were the key considerations?
    Probe: Types of financial, political (including citizen mobilization), or other incentives
  - Who are the actors involved in this initiative? How did you work collectively to design/implement this initiative?
    Probe: Roles and responsibilities of each person and resource mobilization
  - What are the costs (human, material, financial) associated with this initiative?
    Probe: What is the cost of continuity for this initiative?
  - What have been the main challenges encountered in designing/implementing this initiative? And how were they addressed?
- **Institutionalizing the initiatives**
  - How does this initiative fit into the local/regional/provincial institutional landscape? How has your organization interacted with other actors to facilitate its integration into this landscape?
    Probe: What lessons do you learn from these interactions?
  - In your opinion and based on your knowledge, what are the obstacles to the formal institutionalization of this cross‑sectoral initiative? What would help overcome these obstacles?
  - How has COVID‑19 impacted the link between institutional and community networks?
    Probe: How can these ties be strengthened beyond the pandemic?
  - Do you have any other suggestions or comments on how to improve this cross‑sectoral action?

## Abbreviations

CBO, community‑based organizations

COVID‑19, coronavirus disease 2019

IT, information technology

## Data Access

All authors confirm that they had access to the data throughout the research and shared in the writing of the manuscript.
